# Environmental Screening of *Aeromonas hydrophila*, *Mycobacterium* spp., and *Pseudocapillaria tomentosa* in Zebrafish Systems

**DOI:** 10.3791/55306

**Published:** 2017-12-08

**Authors:** Jean-Philippe Mocho, Darren J. Martin, Mollie E. Millington, Yolanda Saavedra Torres

**Affiliations:** ^1^Biological Research Facility, The Francis Crick Institute

**Keywords:** Microbiology, Issue 130, Environmental screening, *Aeromonas hydrophila*, *Mycobacterium* spp., *Pseudocapillaria tomentosa*, zebrafish, *Danio rerio*, health monitoring, biofilm, sludge, quarantine, fish, water microbiology

## Abstract

Health monitoring systems are developed and used in zebrafish research facilities because pathogens of *Danio rerio* such as *Aeromonas hydrophila*, *Mycobacterium *spp., and *Pseudocapillaria tomentosa* have the potential to impair animal welfare and research. The fish are typically analyzed *post mortem* to detect microbes. The use of sentinels is a suggested way to improve the sensitivity of the surveillance and to reduce the number of animals to sample. The setting of a pre-filtration sentinel tank out of a recirculating system is described. The technique is developed to prevent water pollution and to represent the fish population by a careful selection of age, gender, and strains. In order to use the minimum number of animals, techniques to screen the environment are also detailed. Polymerase Chain Reaction (PCR) on surface sump swabs is used to significantly improve the detection of some prevalent and pathogenic mycobacterial species such as *Mycobacterium fortuitum*, *Mycobacterium haemophilum*, and *Mycobacterium chelonae*. Another environmental method consists of processing the sludge at the bottom of a holding tank or sump to look for *P. tomentosa* eggs. This is a cheap and fast technique that can be applied in quarantine where a breeding device is submerged into the holding tank of imported animals. Finally, PCR is applied to the sludge sample and *A. hydrophila* is detected at the sump's bottom and surface. Generally, these environmental screening techniques applied to these specific pathogens have led to an increased sensitivity compared to the testing of pre-filtration sentinels.

**Figure:**
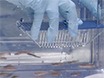


## Introduction

In order to protect research and animal welfare^1^,^2^, the presence of pathogens is monitored within animal facilities. In the case of zebrafish, health monitoring^3^,^4^,^5^,^6^,^7^,^8^,^9^,^10^,^11^ often relies on animals analyzed *post mortem* by histopathology, bacteriology culture, or molecular methods. Testing only colony animals is not the recommended method due to the number of fish and related expenses that would be required to detect pathogens of low prevalence. Therefore, the preferred method is to expose a small group of animals to a higher load of contaminants. These fish are called pre-filtration sentinels. This exposure lasts for months and it involves an increase in the animal carer's workload and/or some purpose-built engineering solution. Another challenge is the screening of imported lines in quarantine where the fertile animals are to be kept alive and this is not compatible with routine assays on carcasses.

We describe here some methods to detect certain zebrafish pathogens (*A. hydrophila, Mycobacterium *spp., and* P. tomentosa*) by screening the aquatic system's environment. The aim is to reduce the number of fish used for health monitoring and to optimize turnover, cost, and sensitivity of the detection. Such methods are an alternative to the use of animals and some techniques can be applied to screening imports in quarantine. For example, Mocho^9^ was able to identify more pathogenic mycobacterial species by performing PCR on sump swabs rather than on zebrafish (including sentinels), and this was obtained with fewer samples. In that same study, *P. tomentosa* eggs were detected with more sensitivity by screening the tank sludge using flotation and microscopy rather than testing fish by PCR and histopathology.

**Table 1** summarizes the various characteristics of sentinel programs^3^,^4^,^5^,^6^,^7^,^8^,^9^,^10^ used by a number of zebrafish facilities. Post-filtration sentinels receive water in the same manner as any colony fish whereas pre-filtration sentinels receive water once it has circulated through colony fish tanks first. For example, pre-filtration sentinels can be set up on the recirculating system by continuously receiving sump water. This may not be an option when there are many independent systems in one room. In this case, one tank of pre-filtration sentinels can be used to screen the whole room. The sentinels are in a static tank, out of the recirculating system, and their water is changed regularly, using only pre-filtration water *i.e.*, sump water from all the systems in the room. This technique is described below as a baseline for comparison with the efficacy of the environmental screening. The proposed set-up is designed to control water quality issues like a decrease of pH or a nitrogen pollution.

The concept of the bacterial environmental screening relies on the hypothesis that bacteria are detectable in biofilm such as that found on the sump wall at the water surface or in the sludge at the bottom of a tank. The sump seems an ideal sampling point in a recirculating aquaculture system since it collects waste (water, feces, feed, and other organic material) from all tanks pre-filtration. The surface of the sump is often easily reachable, the swabbing is fast, and it can be performed aseptically to avoid cross-contamination of the sample (from gloves for example). The concept is used to identify prevalent pathogenic *Mycobacterium* spp. in zebrafish systems^9^,^12^. The technique is described below and we are also reporting detection of *A. hydrophila* in zebrafish sump surface swabs and sludge.

The environmental screening for parasite eggs is based on the detection by Murray *et al.*^13^ and the flotation technique is used routinely for parasitology and microscopic screening of parasite eggs in feces^14^. Mocho^9^ proposed an alternative to the sampling process and showed that the technique could be used to detect other species of the fish biotope. Infected *D. rerio* pass *P. tomentosa* eggs with their feces and the parasite eggs remain at the bottom of the tank, in the sludge. They can be collected there due to their density being greater than water. The density of the eggs is used to process the environmental sample too. A first flotation with centrifugation separates water and light debris from heavier matter. A second centrifugation relies on saturated sugar solution (with a density greater than density of *P. tomentosa* eggs) to allow for the parasite eggs to emerge at the surface of the tube.

The screening for bacteria in the biofilm and for *P. tomentosa* from the bottom of the tank can be combined by performing PCR for all these pathogens on the sludge sample sediment obtained after the first centrifugation. This optimizes the sampling time. The method is described below. We also propose to use these techniques in a quarantine context. To screen imported adult zebrafish that need to be kept alive, a breeding device is inserted to the quarantine tank. After one week, feces and other waste materials in the breeding device are collected and screened by microscopy or PCR. The technique is described below and some *P. tomentosa* eggs were detected by microscopy in this context.

## Protocol

### 1. Exposure of Pre-filtration Sentinels Out of a Recirculating System

1. **Set a clean 8 L tank out of a recirculating system. Fill it up with water coming from the sumps. Add 2 ceramic beads or sponge cubes of bio-media from the systems to screen (Figure 1). Add 1 - 2 *D. rerio*/L (*i.e.*, 12 fish). Use wild type fish of the dominant genetic background in the facility, for example AB.**
  1. Select at least 6 fish as follows: at least one female and one male below 6 months of age, one female and one male between 6 and 18 months, and one female and one male above 18 months of age.
2. **Feed once a day. Vary the diets (*e.g.*, dry diet, brine shrimp) to make sure the sentinels are exposed to all the diets used in the zebrafish facility. Change water on Monday, Wednesday, and Friday, *i.e.*, three times a week for 4 months.**
  1. To conduct the water change, transfer sentinels and bio-media into a temporary tank. Empty completely the sentinel tank and clean it. Refill the sentinel tank with sump water only. Put the sentinels and bio-media back.
3. Expose the sentinels for 4 months to sump water. Euthanize the fish by an approved method such as immersion in an overdose solution of 2-phenoxyethanol (3 mL/L).
4. **To confirm death, wait for 10 min after cessation of opercula movement.**
  1. Grab the cadaver with forceps and freeze it entirely at -80 °C in an identified container. This will be used for PCR^12^,^15^.
  2. Alternatively, cut the tailat the caudal peduncle, nick the abdominal wall, and set in 4% formalin. CAUTION: Use gloves and fume cabinet for histopathology. Label the sample container.

### 2. Sump Swabs

1. Use a sterile dry swab with a plastic shaft. Wear gloves. Locate the surface to swab (sump wall at the surface of the water) and remove any item preventing easy access to the surface. Choose a sump surface with low flow.
2. **Unsheathe the swab by removing the outer packaging and expose the sterile cotton tip to the air. Avoid cross contamination of the swab by taking care not to touch untested surfaces.**
  1. Swab the sump wall over 5 - 10 cm to absorb the water and biofilm at the sump water surface level.
  2. Sheath the swab back or break the tip into a sterile centrifuge tube. Label the sample and send for PCR testing or freeze at -80 °C.

### 3. Detection of *P. tomentosa* Eggs at the Bottom of a Tank

1. **Sludge analysis by microscopy**
  1. Use a 60 mL syringe^9^ to aspirate the sludge at the bottom of a sump or any tank holding fish including sentinels. Divide the sample into 15 mL tubes. Close the tubes with their screw tops and label the tubes.
  2. Prepare the sugar saturated solution (specific gravity = 1.27) by mixing 227 g of granulated sugar in 177 mL of hot water with a magnetic stirrer^14^.
  3. Centrifuge the 15 mL tubes at 175 - 250 x g for 10 min in a centrifuge with swing buckets. Decant the tubes and keep the sediment in their tube.
  4. Fill up the tubes halfway with the sugar saturated solution. Close the tubes with their screw top and thoroughly mix the sediment with the solution.
  5. Place the tubes in the centrifuge swing buckets and fill them up with sugar saturated solution to the top. Set one cover glass gently on top of each tube and in contact with the sugar saturated solution.
  6. Centrifuge at 175 - 250 x g for 10 min. Note that some cover glass may fall and break during centrifugation hence there are 4 tubes for each 60 mL sample. Lift the cover glass and set it on a glass slide. Label the slide with a pencil or marker pen.
    1. In case too many cover glass breakages occur, fill up most of the tube with the sugar saturated solution, centrifuge at 175 - 250 x g for 10 min, fill up to the top with the sugar saturated solution, then gently set the cover glass. Wait for 30 min.
  7. Look for the *P. tomentosa* eggs with the microscope^13^ (Figure 2 and **Video 1**). Identify the bipolar plugs at magnification 400X^6^. NOTE: The size of the eggs is 57 - 78 µm long and 27 - 39 µm diameter^16^,^17^. Note that one positive slide is enough for the 60 mL sample to be declared positive.
2. **Screening imported animals in quarantine for *P. tomentosa* eggs**
  1. Set male and female *D. rerio* in a tank. Add to the tank a device normally used to harvest and preserve spawned eggs but use it here to collect feces (Figure 3). NOTE: For example, a full 1 L breeding tank (outer tank and inner tank with grated bottom) is completely submerged in a 13 L tank, allowing free access for the fish to move in and out of the breeding device.
  2. Remove the breeding device after one week and harvest the collected sludge in the breeding device as described in step 3.1 "sludge analysis by microscopy."

### 4. PCR on Sludge Sediment

1. Aspirate the sludge at the bottom of a tank or sump with a 60 mL syringe^9^ and transfer the sample to a 60 mL tube. Close the tube with its screw top and label the tube. Dispose of the syringe.
2. Shake the 60 mL tube and transfer 15 mL to a 15 mL tube. Close the tube with its screw top and label the tube.
3. Centrifuge the 15 mL tube at 175 - 250 x g for 10 min in a centrifuge with swing buckets. Decant the tubes and keep the sediment in the tube.
4. **Unsheathe a swab by removing the outer packaging and expose the sterile cotton tip to the air. Avoid cross contamination of the swab by taking care not to touch untested surfaces.**
  1. Swab the sediment in the tube for 15 s.
  2. Sheath the swab back or break the tip into a sterile centrifuge tube. Label the sample, freeze at -80 °C and send for PCR testing. NOTE: 45 mL remain in the 60 mL tube. This can be kept for detection of *P. tomentosa* eggs by sludge analysis by microscopy as described in step 3.1, for example, to confirm the PCR result. The PCR in the sludge can be tried for screening imported animals following step 3.2.

## Representative Results

The benefits of the sump swabs to identify prevalent *Mycobacterium* spp. compared to fish samples are supported by the results in Figure 4. Of 115 fish tested, *M. chelonae* and *M. haemophilum* were detected in 5% and 3% of the samples, respectively. No other pathogenic mycobacterial species was identified. From the same systems, 49 sump swabs revealed the presence of 5 mycobacterial species. Odds ratio are calculated with the hypothesis that *M. chelonae* and *M. fortuitum* are detected more frequently by PCR in the surface sump swabs than in the fish sample. This is statistically significant with respective odds ratio of 11 (95% CI: 4 to 29; *p* <0.0001) and 306 (95% CI: 18 to 5208; *p* = 0.0001). The results show that the surface sump swab technique is a valuable alternative to the sole use of sentinels to screen the zebrafish facility for *Mycobacterium *spp. The environmental samples were also used to screen for *A. hydrophila*. These bacteria were detected in fish, sludge, and surface samples (Figure 4). This also supports the ability of the proposed techniques to screen the fish biotope.

Regarding the sludge analysis to detect *P. tomentosa* eggs, Mocho^9^ detected the parasite in 27% of the fish samples by PCR and histopathology whereas the eggs were detected in 93% of analyzed sludge from the same system. Here, the technique was challenged to reproduce quarantine screening. In this context, imported animals cannot be sampled, and assessing their health status in a timely manner helps with the biosecurity management. Fish of unknown health status from a *P. tomentosa *positive facility were set in 8 tanks with breeding devices: maximum of 16 fish/13 L tank, 7 transgenic and 1 wild type line, mixed gender, aged 4 - 24 months. The sludge was harvested from the devices after one week and analyzed by microscopy. *P. tomentosa* eggs were seen in 7 (88%) samples. Finally, the PCR detection of the parasite was trialed on sump swabs and sludge sediments. 4 out of 6 sludge samples were PCR positive and all results were negative for the surface swabs. This is not surprising since the sludge screening relies on the ability of the eggs to fall to the bottom of the tank. This shows that the sludge analysis techniques can be used to screen *D. rerio* aquaria for *P. tomentosa* infestation and that the methods can be adapted for the screening of imported animals.

**Figure:**
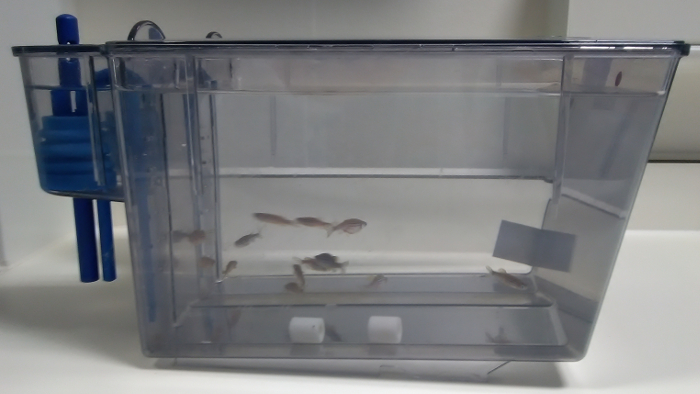

**Figure 1: Pre-filtration sentinel tank out of the recirculating system.** An 8 L tank is filled up with water and bio-media from the sumps of the systems to screen. The two white ceramic bio-media beads sit at the bottom of the tank (in the middle of the picture). 12 fish are selected according to their age, strain, and gender, and they are added to the sentinel tank. Please click here to view a larger version of this figure.

**Figure:**
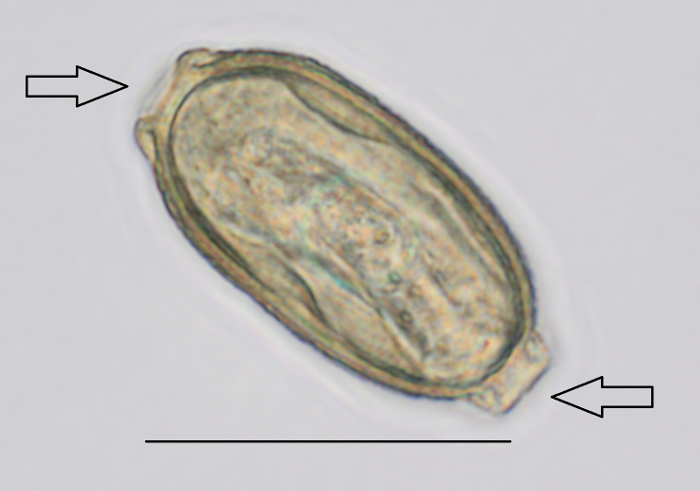

**Figure 2: *P. tomentosa* egg detected during sludge analysis by microscopy. **Magnification used was 400X. Arrows indicate the bipolar plugs. Scale bar = 50 µm. Please click here to view a larger version of this figure.

**Figure:**
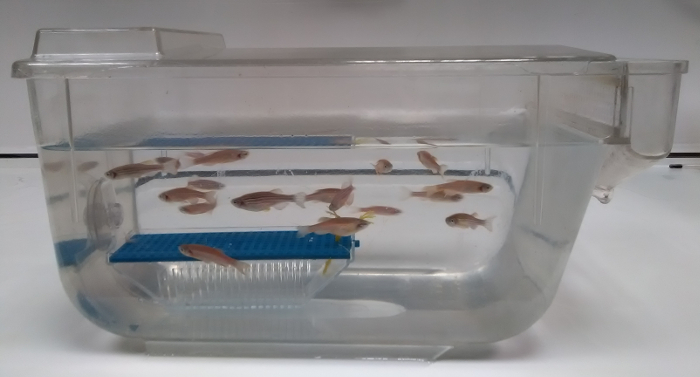

**Figure 3: Breeding device submerged in a fish holding tank to collect sludge for analysis.** This tank is set on a bench for the purpose of the picture; it is set otherwise in the recirculating system. Please click here to view a larger version of this figure.

**Figure:**
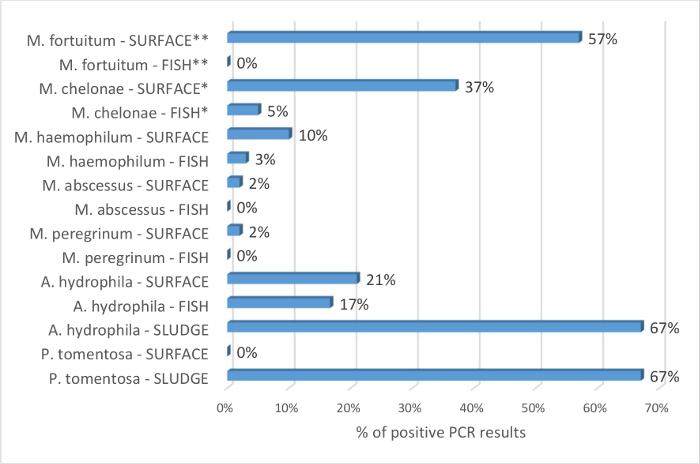

**Figure 4: Percentage of identification of *Mycobacterium* spp., *A. hydrophila*, and *P. tomentosa* by PCR on fish, surface sump swabs, and sump sludge.** Percentage is obtained by dividing the number of positive results for each pathogen species by the number of tested samples. The percentage of positive results is given by sample type as FISH, SURFACE, or SLUDGE, and indicated after the bacteria's name. 115 fish and 49 surface sump swabs were tested by PCR for identification of *Mycobacterium* spp. The data are compiled with Mocho's^9^ results since it is an extension of this study. The fish are mainly pre-filtration sentinels as per protocol section 1. Rarely, when sentinels were not available, escapees and old colony fish (>18 months) were sampled. All tested systems for *Mycobacterium* spp. were tested on fish and sump swabs. Odds ratio are calculated with the hypothesis that *M. chelonae* and *M. fortuitum* are detected more frequently by PCR in the surface sump swabs than in the fish sample. This is statistically significant with respective odds ratio of 11 (95% CI: 4 to 29; *p* <0.0001) and 306 (95% CI: 18 to 5208; *p *= 0.0001). *Mycobacterium marinum* PCR was negative for all the samples and it is therefore deemed absent from these facilities and not included in the analysis. 12 fish, 14 surface sump swabs, and 6 sump sludge were tested by PCR for the presence of *A. hydrophila*. 6 sumps were tested for the presence of *P. tomentosa* on the surface and in the sludge. PCR = Polymerase Chain Reaction. CI = Confidence Interval. * and ** indicate statistical significance; other comparisons were not statistically significant. Please click here to view a larger version of this figure.


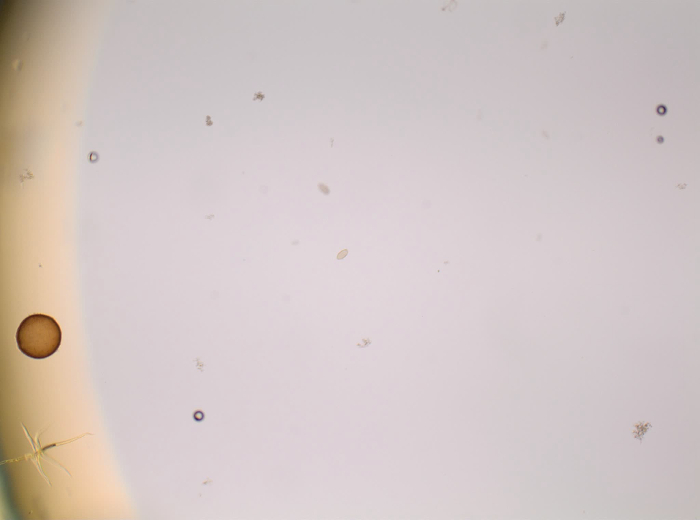
**Video 1: Scanning for an egg of *****P. tomentosa***** with the microscope.** The slide is obtained from sludge analysis as described in step 3.1. The field is scanned to detect an egg of *P. tomentosa*. Once a structure is recognized, it is zoomed in to confirm the identification at higher resolution. Please click here to view this video. (Right-click to download.)

**Table:** 

**Authors**	**Age at the start of exposure**	**Length of exposure**	**Sampling age**	**Pre or Post filtration**	**Gender**	**Strain(s)**
Barton *et al.*^3^	4 months	6 months	10 months	Pre-filtration	N/A	N/A
Borges *et al.*^4^	6 months	6 months	12 months	Pre-filtration	N/A	AB wild type
Collymore* et al.*^5^	As young as possible	3 months	<6 months	Pre-filtration	N/A	N/A
Geisler *et al.*^6^	4 months	4 months	8 months	Pre and post filtration	N/A	AB wild type
Liu* et al.*^7^	3 months	1-6 months	4-9 months	Pre and post filtration	N/A	Wild type
Martins* et al.*^8^	3 months	3 months	6 months	Pre-filtration	N/A	Wild type
Mocho^9^	<6 months, 6 - 12 months, >18 months	4 months	7 - 24 months	Pre-filtration	1 female and 1 male of each age group	AB wild type
Murray *et al.*^10^	3-4 months	3 months, 6 months, 1 year	7, 10, and 16 months	Pre and post filtration	N/A	AB wild type

**Table 1: Comparison of sentinel settings in zebrafish facilities.** The sentinel fish may be selected according to their age, gender, or strain. They are exposed for a defined period and they receive pre- or post-filtration system water. The data are compiled from the 2016 special issue on health of the Zebrafish journal^3^,^4^,^5^,^6^,^7^,^8^,^9^,^10^.

## Discussion

### Limitations of the Techniques, Critical Steps, and Troubleshooting:

The age, gender, strain, and length of exposure of the sentinels are not standardized. This is shown in **Table 1**. There is very little screening of fish below 6 months of age, or of aged fish. There may be some pathogens that affect the young fish as there are some pathogens that are more prevalent in the older population^10^,^18^,^19^,^20^. Similarly, the gender is not considered in the selection of some sentinel groups despite some report that there is a gender bias for some pathogens^21^. The proposed technique tries to address these issues, although the choice of the strain could be made according to a specific pathogen to monitor. For example, TU could help with the detection of *Mycobacterium* spp.^12^,^22^, but there is a risk that the sentinels would then act as a reservoir or display clinical signs. Regarding the length of exposure, the approach of the Zebrafish International Resource Center^10^ increases the chances to detect pathogens that could be missed with an inadequate contamination period. The need for prolonged exposure implies that sentinels are not readily available. The addition of the environmental samples allows some flexibility and the multiplication of the screening events. For example, sampling can take place every other month with a 4 month interval between each screening method. This may reduce the lapse of time before a newly introduced pathogen is detected.

The environmental screening techniques rely on the detection of pathogens in the environment. The pathogens are shed by the fish and therefore diluted in the system water. The possibility of capturing the pathogens by water filtration^23^ was not explored. The methods we describe are only effective if pathogens are given enough time to multiply in fish and biofilm to reach a threshold of contamination allowing detection. This limitation of the techniques is minimized by a critical selection of the sampling sites: the sludge in the tank is sampled rather than the sump sludge, and the water and biofilm are sampled at the surface of the sump rather than in a tank or post-filtration. Nonetheless, all the samples from the same system are unlikely to give the same results. Positive results for *P. tomentosa* can be confirmed by using another assay (histopathology, PCR, or sludge analysis). Mycobacterial PCR positive results can be confirmed by culture or by another diagnostic laboratory. However, when establishing a health status, further samples are recommended to confirm negative results from any environmental screening technique.

### Significance of the Technique with Respect to Existing/Alternative Methods:

*Mycobacterium* spp. are common in the environment and their presence in the sump does not predict their pathogenicity^12^. Mocho^9^ showed that monitoring mortality rates is key to survey the developments of health issues. The use of animal samples remains essential to any veterinary investigation. Health monitoring implies detection of all prevalent pathogens in a facility and this cannot be achieved with the sole use of environmental screening techniques. Nevertheless, a lack of sensitivity of the diagnosis tools can delay or prevent an accurate description of the health status. Whilst the use of sentinels reduces the number of fish required to detect a prevalent microbe in the population, the lack of sensitivity adds weight to using a combination of methods, including environmental screening^5^,^23^. Indeed the Specific Pathogen Free status is usually defined as the absence of a species in the facility such that environmental and animal samples must test negative^24^,^25^.

The sump swab results to identify *Mycobacterium *spp. show that relying on fish samples may lead to a false negative health status. The 6 tested mycobacterial species are described as pathogenic or potential pathogenic in zebrafish^15^ and some would not be eliminated by egg surface disinfection with chlorine^26^ as routinely performed in quarantine. Therefore, the false negative may have some consequences for collaborators who import lines. For example, *M. fortuitum* was missed by the PCR on fish sample but more than half of the sump swab PCR detected it. Considering that these mycobacteria are more resistant to chlorine than others and their ability to grow in the water systems^27^, it is a risk for the non-contaminated importing facility. To allow the import of lines, managers need to trust and compare the health reports of the exporting facility with theirs. The ICLAS Performance Evaluation Program^28^ is key to that process in rodents. The RESAMA network reports the detection of *M. gordonae* and *M. mucogenicum* in French *D. rerio*^11^. These Mycobacteria are not proposed in the panels of the commercial laboratories that we use. It would be useful to extend the ICLAS program and to harmonize the diagnostic assays as well as the list of pathogenic species^29^.

*A. hydrophila* is also a pathogen that has the potential to be introduced when importing animals, although its susceptibility to chlorine^30^ makes its elimination more likely during routine egg surface disinfection. The sump, swab, and sludge results show that environmental screening can be used to detect this pathogen. Other bacteria like *Mycobacterium* spp. have been detected in the sludge by PCR^23^. This type of sample is particularly relevant since it allows the detection of shed pathogens. For example, another new application is the sludge analysis to screen imported fish in quarantine for *P. tomentosa*. The parasite is a threat to animal's health^13^ and neoplasia models^16^. Moreover, chlorine concentrations used in routine zebrafish egg surface disinfection are not efficient^31^. Therefore, the ability to screen the imported animals with a one-week turnover and without any fish euthanasia seems very attractive. This technique can influence the quarantine and biosecurity rules by allowing a triage of imports. A decision-making process is then designed according to the prevalent pathogens in the exporting facility, the detected pathogens in the samples from the imported fish, and the risk of compromising the health status of the importing facility^10^.

### Future Applications or Directions after Mastering These Techniques:

Even if routine quarantine treatment is the chosen option, the efficacy of such medication^32^,^33^,^34^,^35^,^36^ can be assessed with the breeding device sludge analysis. More generally, the environmental screening could be used to test compounds against bacteria and parasite eradication, including in the fish biotope. Another niche application of the environmental screening is to monitor the pathogen population in the live feed^37^,^38^. Though the main application of these techniques is as a valuable addition to the diagnosis toolbox for the health monitoring in zebrafish facilities. Thanks to a more accurate, cost and time efficient definition of the health status, sump swabs and sludge analysis are complementary to the sentinel surveillance and the routine quarantine practice. Indeed, the future of these techniques is to be a routine part of any aquatic laboratory health report.

## Disclosures

The authors have nothing to disclose.
